# ROCK Inhibitor Y-27632 Suppresses Dissociation-Induced Apoptosis of Murine Prostate Stem/Progenitor Cells and Increases Their Cloning Efficiency

**DOI:** 10.1371/journal.pone.0018271

**Published:** 2011-03-28

**Authors:** Li Zhang, Joseph M. Valdez, Boyu Zhang, Lei Wei, Jiang Chang, Li Xin

**Affiliations:** 1 Department of Molecular and Cell Biology, Baylor College of Medicine, Houston, Texas, United States of America; 2 Department of Pediatrics, Indiana University School of Medicine, Indianapolis, Indiana, United States of America; 3 Institute of Biosciences and Technologies, Texas A & M Health Science Center, Houston, Texas, United States of America; 4 Department of Pathology, Baylor College of Medicine, Houston, Texas, United States of America; 5 Dan L. Duncan Cancer Center, Baylor College of Medicine, Houston, Texas, United States of America; SanfordBurnham Medical Research Institute, United States of America

## Abstract

Activation of the RhoA/ROCK signaling pathway has been shown to contribute to dissociation-induced apoptosis of embryonic and neural stem cells. We previously demonstrated that approximately 1 out of 40 Lin^−^Sca-1^+^CD49f^high^ (LSC) prostate basal epithelial cells possess the capacities of stem cells for self-renewal and multi-lineage differentiation. We show here that treating LSC cells with the ROCK kinase inhibitor Y-27632 increases their cloning efficiency by 8 fold in an *in vitro* prostate colony assay. Y-27632 treatment allows prostate colony cells to replate efficiently, which does not occur otherwise. Y-27632 also increases the cloning efficiency of prostate stem cells in a prostate sphere assay and a dissociated prostate cell regeneration assay. The increased cloning efficiency is due to the suppression of the dissociation-induced, RhoA/ROCK activation-mediated apoptosis of prostate stem cells. Dissociation of prostate epithelial cells from extracellular matrix increases PTEN activity and attenuates AKT activity. Y-27632 treatment alone is sufficient to suppress cell dissociation-induced activation of PTEN activity. However, this does not contribute to the increased cloning efficiency, because Y-27632 treatment increases the sphere-forming unit of wild type and Pten null prostate cells to a similar extent. Finally, knocking down expression of both ROCK kinases slightly increases the replating efficiency of prostate colony cells, corroborating that they play a major role in the Y-27632 mediated increase in cloning efficiency. Our study implies that the numbers of prostate cells with stem/progenitor activity may be underestimated based on currently employed assays, supports that dissociation-induced apoptosis is a common feature of embryonic and somatic stem cells with an epithelial phenotype, and highlights the significance of environmental cues for the maintenance of stem cells.

## Introduction

The Rho family of small GTPases are critical mediators that regulate a plethora of cellular processes including cellular polarity, motility, proliferation and apoptosis [1], [2]. A major downstream effector for Rho GTPases is the ROCK serine/threonin kinase (Rho-associated, coiled-coil-containing protein kinase), which consists of two family members ROCK I (P160ROCK) and ROCK II with redundant functions [3], [4]. ROCK controls actin-cytoskeleton assembly and cell contractibility by phosphorylating numerous downstream target proteins [3], such as the regulatory myosin light chain (MLC) and the actin-binding LIM kinases. Consequently, ROCK mediates membrane blebbing, enhances actin-myosin contraction, and activates caspase signaling cascades and cellular apoptosis.

A peculiar feature of human embryonic stem cells is their propensity for dissociation-induced apoptosis, which used to be a technical obstacle for genetic manipulation of those cells [5]. Recent work by Ohgushi *et al* and Chen *et al* showed that this dissociation-induced apoptosis is due to the Rho-ROCK pathway-mediated actomyosin hyperactivation [6], [7]. This explains why the selective ROCK inhibitor Y-27632 is capable of increasing survival and cloning efficiency of dissociated single human embryonic stem cells [8]. Ohgushi *et al* further showed that epiblast-derived mouse embryonic stem cells also succumbed to dissociation-induced apoptosis through ROCK/Myosin activation, suggesting that dissociation-induced actomyosin hyperactivation is a common phenomenon in vertebrate embryonic ectodermal cells [6]. Recently, it was reported that inhibition of Rho/ROCK pathway by Y-27632 also enhances *in vitro* survival of mouse ES cell derived neural precursors [9], mouse intestinal stem cells [10] and human keratinocytes [11]. These studies imply that dissociation-induced Rho/ROCK-mediated apoptosis is a common feature of stem/progenitor cells with an epithelial phenotype, irrespective of their embryonic layer origin.

Prostate epithelia are of endodermal origin [12]. There are three epithelial cell types in the prostate: the secretory luminal cell, basal cell and a very rare neuroendocrine cell [13]. We and others have demonstrated that a small fraction of adult murine and human prostate basal cells are capable of forming 2-dimensional colonies or 3-dimensional serially-passagable spheroids *in vitro* and regenerating prostate tissues composed of multiple cell lineages *in vivo* [14], [15], [16], [17], [18]. These works demonstrate that those basal epithelial cells possess the stem cell capacities for self-renewal and multi-lineage differentiation. We calculated that approximately 1 out of 40 basal cells possess stem cell activity [15]. Since prostate epithelial cells are dissociated into single cells before being cultured in those assays, we reasoned that the frequency of the cells that possess stem cell activity could be underestimated if prostate stem cells are also vulnerable to dissociation-induced apoptosis. In this study, we demonstrated that murine prostate epithelial stem cells are indeed susceptible to dissociation-induced apoptosis. Y-27632 treatment suppresses apoptosis and increases the cloning efficiency of prostate stem cells. Our work supports that dissociation-induced apoptosis is a common feature for embryonic and somatic stem cells with an epithelial phenotype.

## Materials and Methods

### Mouse strains

The wild type C57BL/6, FVB, NOD.CB17-*Prkdc^scid^*/J, B6.Cg-Tg(ACTB-DsRed*MST)1Nagy/J and PTEN^flox/flox^ transgenic mice were purchased from the Jackson Laboratory (Bar Harbor, ME). The *ARR2PB-Cre* transgenic mice were from Dr. Fen Wang at the Institute of Bioscience and Technology, Texas A&M Health Science Center. The *ROCK1* null transgenic mice have been characterized previously [19]. All animals used in this study received humane care in compliance with the regulations relating to animals and experiments involving animals and adheres to principles stated in the Guide for the Care and Use of Laboratory Animals, NIH Publication, 1996 edition, and the protocol (AN-4938) was approved by the Institutional Animal Care Committee of Baylor College of Medicine.

### FACS

Dissociated murine prostate cells were suspended in DMEM/10% FBS and stained with antibodies for 15 min at 4°C. The antibodies used were biotin- or FITC-anti CD31,CD45 and Ter119 antibodies (eBioscience, San Diego, CA), FITC- or PE-anti Sca-1 antibody (eBioscience, San Diego, CA), Alexa 647-anti CD49f antibody (Biolegend, San Diego, CA) and strepavidin-Alexa 750 (Invitrogen, Carlsbad, CA). FACS analyses and sorting were performed by using the BD LSR II and Aria II, respectively (BD Biosciences, San Jose, CA).

### Prostate cell colony assay, prostate sphere assay and prostate regeneration assay

Prostate colony and sphere assays were performed as previously described [17]. Dissociated prostate epithelial cells were prepared as described previously from 7–12 week old mice of different genetic backgrounds mentioned in the text. Dissociated cells were cultured on petridishes coated with type I rat tail collagen, matrigel or mitomycin (Roche Applied Science, Indianapolis, IN)-treated Swiss 3T3 cells (kindly provided by Dr. Feng Yang at Baylor College of Medicine) in PrEGM media (Lonza, Walkersville, MD). In some of the assays, Y-27632 (Stemcell Technologies Inc, Vancouver, Canada, or Enzo Life Sciences, Plymouth Meeting, PA) was added in the media at a final concentration of 10 µM. The prostate sphere assay and the dissociated prostate cell regeneration assay were performed as described previously [17], [20].

### RNA interference

Small interfering RNA (siRNA) duplex targeting murine ROCK2 were selected based on previous publication [21]. The siRNA target sequences were 5′-CAGAAGCGTTGTCTTATGCAA-3′ and 5′-TTGGATAAACATGGACATCTA-3′. A scrambled siRNA was used as the control and the sequence was 5′-GGAACCAATTTCGGTAATAAT-3′. Murine prostate epithelial cells were transfected with these siRNAs at a final concentration of 20 nM using HiPerfect transfection reagent (Qiagen, MD, USA). Cells were collected for the Western Blot and colony forming assays 48 hours post-transfection.

### Western Blot analysis

Total protein was extracted with RIPA buffer (20 mM Tris-HCl, pH 7.5, 150 mM NaCl, 1 mM Na_2_EDTA, 1 mM EGTA, 1% NP-40, 1% sodium deoxycholate, 2.5 mM sodium pyrophosphate, 1 mM β-glycerophosphate, 1 mM Na_3_VO_4_, and protease inhibitors). Protein concentrations were determined by Bradford Assay kit (BioRad, Hercules, CA). Protein was separated by 8% or 12% SDS/PAGE and transferred onto a PVDF membrane (Amersham Biosciences, Arlington Heights, IL). The membrane was blocked in 5% skim milk, and subsequently incubated with primary antibodies against β-actin (Sigma, St. Louis, MO), ROCK1 (Santa Cruz Biotechnology, Santa Cruz, CA), ROCK2 (BD Biosciences, Two Oak Park, Bedford, MA), MYPT, phospho-MYPT Thr853, Cleaved caspase 3, PTEN, phospho-PTEN Thr-380, AKT, phospho-AKT Thr473 (Cell Signaling Technology, Beverly, MA) at 4°C overnight followed by incubation with peroxidase-conjugated goat anti-mouse IgG or goat anti-rabbit IgG (Jackson ImmunoResearch, Inc., West Grove, PA), and developed with Pierce ECL reagent (Thermal Scientific, Rockford, IL).

### Annexin V staining

Primary murine prostate cells were cultured in PrEC media in 6-cm Petri dishes with 10 µM Y-27632 for 6 days till cells were approximately 30–40% confluent. Media was replaced and cells were cultured in PrEC with or without 10 µM Y-27632 for another 24 hours. Cells were dissociated and cultured in PrEGM media in low-attachment plates for 2 hours with or without 10 µM Y-27632. Cells were washed twice with cold PBS and then resuspended in 100 µl Annexin V Binding Buffer (Biolegend, San Diego, CA). 5 µl of Alexa Fluor® 647 Annexin V (Biolegend, San Diego, CA) and 2 µl of 50 µg/ml PI solution (Biolegend, San Diego, CA) were added. Cell were stained for 15 min at RT (25°C) in the dark and analyzed by flow cytometry.

### Cell death assay

Histone-associated DNA fragments were measured using the Cell Death Detection ELISA Kit (Roche Applied Science, Indianapolis, IN). Lysates were collected from 6,000 cells that were dissociated and cultured in suspension in ultra-low attachment plates in PrEGM media for 4 hours with or without 10 µM Y-27632. Assays were performed according to the manufacturer's instructions.

## Results

### Y-27632 treatment increases the in vitro prostate cell colony-forming activity

An *in vitro* prostate cell colony assay was previously used as a surrogate assay to measure prostate stem cell activity [15], [22]. In this assay, dissociated prostate epithelial cells are cultured on top of irradiated or mitomycin-treated fibroblast feeder layer cells in low-calcium, serum-free media containing growth factors. A small fraction of prostate epithelial cells form clonogenic colonies containing cells that are mostly dual positive for the basal cell marker keratin 5 and the luminal cell marker Keratin 8. We previously demonstrated that a small fraction (2–4%) of the Lin^−^Sca-1^+^CD49f^high^ basal cells are the cells that form these colonies [15]. Since prostate epithelial cells are detached from their normal environmental cues and manipulated in suspension in this assay, we wondered whether the dissociation-induced, Rho/ROCK activation-mediated actin-myosin hyperactivation would cause apoptosis of dissociated prostate stem cells, leading to an underestimation of the percentage of prostate stem cells. To test this hypothesis, we sought to determine whether inhibiting ROCK activity by Y-27632 would increase prostate cell colony-forming activity in this assay.

**Figure 1:**
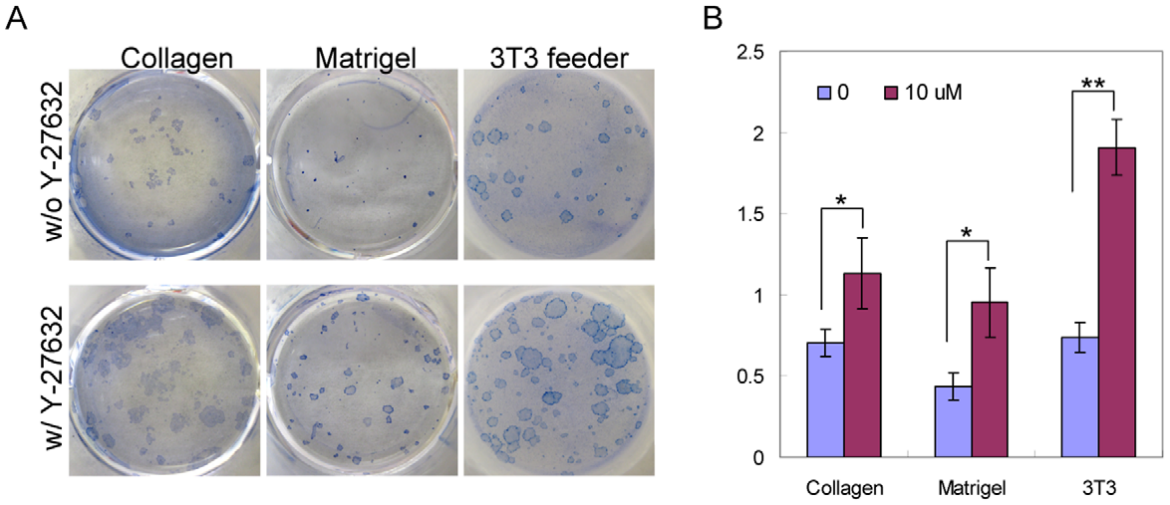
Y-27632 treatment increases the cloning efficiency of prostate stem cells in an in vitro prostate colony assay. (A) Trypan blue staining of prostate colonies grown in 12-well plates coated with collagen, matrigel and mitomysin-treated Swiss3T3 feeder layer with or without Y-27632. (B) Bar graphs compare the colony-forming activity. *P<0.05, **P<0.0005.

Dissociated single prostate cells were prepared from 2-month-old male DsRED transgenic mice. These cells are fluorescently marked due to the expression of the red fluorescent protein driven by the beta-actin promoter. Dissociated cells were seeded on top of mitomycin-treated Swiss 3T3 fibroblast feeder cells at a density of 5000 cells per well in a 12-well plate in triplicates without Y-27632 or with 10 µM Y-27632. Y-27632 at this concentration is generally agreed to specifically inhibit ROCK kinase [3]. Colonies formed after a seven-day incubation and were enumerated under a fluorescent microscope before being fixed and further stained with trypan blue (Fig. 1A). Fig. 1B shows that the percentage of the cells that form colonies in the Y-27632 group is approximately 2.5 fold of that in the control group (mean CFU is 0.74±0.09% for the control group and 1.90±0.17% for the Y-27632 group, n = 3, Student's *t* test was performed, *P* = 0.00047).

Stem cells have also been shown to be able to grow on Type I collagen- or matrigel-coated plates [15], [23], [24], [25]. To investigate whether the Y-27632 induced increase in colony forming activity is dependent on cellular attachment to specific substrata, we performed experiments under the same conditions except that cells were cultured on collagen or matrigel-coated plates. Fig. 1 shows that more colonies formed in the Y-27632 group irrespective of the plate coating. The numbers of formed colonies in the Y-27632 group is approximately 1.6 and 2.2 fold of those in the control group in the collagen and matrigel groups, respectively (mean CFU is 0.71±0.08% for the control group and 1.13±0.22% for the Y-27632 group when using collagen-coated plates, n = 3, *P* = 0.035; mean CFU is 0.43±0.08% for the control group and 0.95±0.21% for the Y-27632 group when using the matrigel-coated plate, n = 3, *P* = 0.017). These data demonstrate that Y-27632 treatment increases the number of the cells that can form colonies in the prostate cell colony assay.

### LSC basal cells contribute to the increased colony-forming activity by Y-27632 treatment

**Figure 2:**
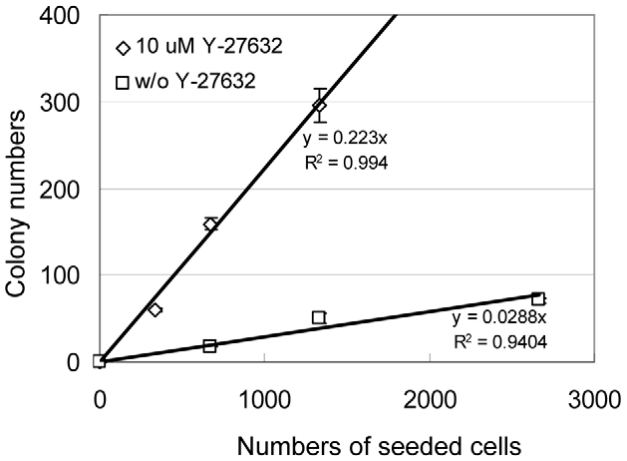
Y-27632 treatment increases the prostate colony-forming activity of LSC (Lin^−^Sca-1^+^CD49f^high^) basal cells by 8 fold. FACS sorted LSC cells from dsRED mice were plated on mitomysin-treated Swiss3T3 layer cells in a dilution series ranging from 133 to 2,667 cells per well with and without Y-27632. Graph shows the number of colonies grown at each dilution plotted versus the input cell number. The slopes represent colony-forming units.

We previously showed that LSC basal cells possess almost all the colony-forming activity in the prostate colony assay [15]. The prostate colony cells in the control and Y-27632 treated groups are phenotypically similar since they both express the basal cell marker keratin5 (K5) and the luminal cell marker keratin8 (K8) simultaneously (data not shown). To definitively investigate whether the increased colony-forming activity is still from LSC basal cells, or alternatively is from luminal cells that were previously incapable of forming K5^+^K8^+^ colonies, we FACS sorted LSC cells and luminal cells and cultured them in serial dilution in the colony assay in duplicates with or without Y-27632. After a seven-day incubation, a few colonies grew out from the FACS-sorted luminal cells in both the control and the Y-27632 groups (data not shown). In contrast but as expected, many colonies formed from the LSC basal cells. As shown in Fig. 2, while only approximately 2.9% LSC basal cells were able to form colonies in the absence of Y-27632, almost 22.3% LSC basal cells formed colonies in the presence of Y-27632. These results demonstrated that upon Y-27632 treatment the increased colony-forming activity is from the LSC basal cells.

### Y-27632 treatment increases the replating efficiency of prostate colony cells

**Figure 3:**
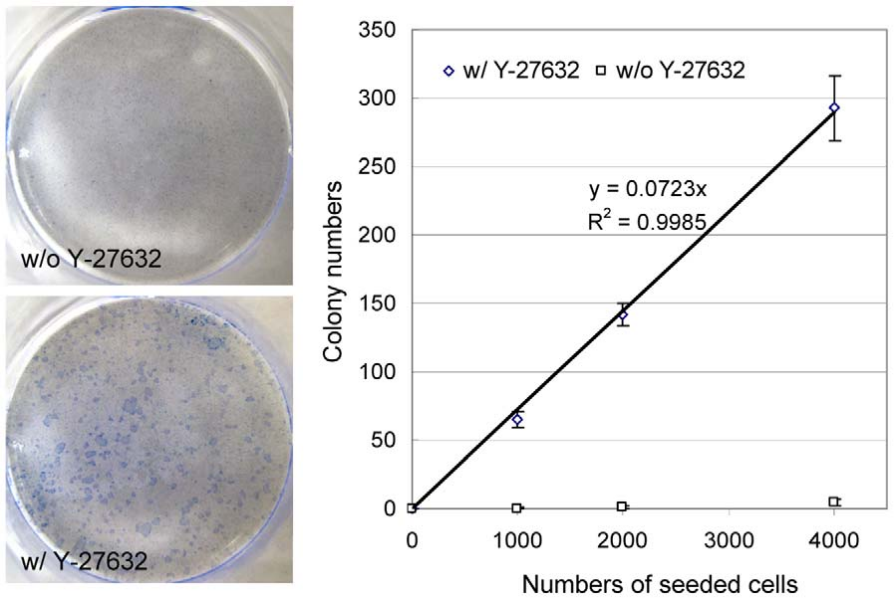
Y-27632 treatment confers prostate colony cells with replating capacity. Primary prostate colony cells were dissociated by trypsin and plated on collagen-coated 12-well plates in a dilution series ranging from 1,000 to 4,000 cells per well with and without Y-27632. (A) Trypan blue staining of secondary prostate colonies in the absence and presence of Y-27632. (B) Graph shows the number of colonies grown at each dilution plotted versus the input cell number. The slopes represent colony-forming units.

An intriguing issue with the colony assay is that after dissociation, few primary colony cells can replate and form secondary colonies [25]. This phenomenon has negated the application of this assay as a *bona fide* stem/progenitor cell assay, since these cells seem to lack self-renewal activity. We sought to determine whether ROCK activation inhibits the ability of prostate colony cells to replate. Primary prostate colonies from DsRED transgenic mouse prostate cells were trypsinized and dissociated into single cells. Dissociated cells were serially diluted and replated on mitomycin-treated swiss 3T3 feeder cells in duplicates with or without Y-27632. Fig. 3 shows that in the absence of Y-27632 almost no colonies grew out even when 4000 cells were plated. In contrast, approximately 7% of dissociated prostate colony cells are capable of generating secondary prostate colonies in the Y-27632 treated group (Fig. 3). The average sizes of secondary colonies are smaller compared with primary colonies after a 7-day culture.

### Y-27632 treatment also increases stem cell activity in the prostate sphere assay and the dissociated prostate cell regeneration assay

We previously established two other functional assays to measure stem cell activity in the prostate. In an *in vitro* prostate sphere assay, some prostate epithelial cells are capable of forming serially-passagable clonogenic spheroid structures when cultured inside semi-solid matrigel in the presence of low-calcium serum-free media supplemented with growth factors [17]. In an *in vivo* dissociated prostate cell regeneration assay, a small fraction of cells are capable of forming clonogenic globular structures that are composed of all three prostate epithelial cell lineages [14], [15], [20].

**Figure 4:**
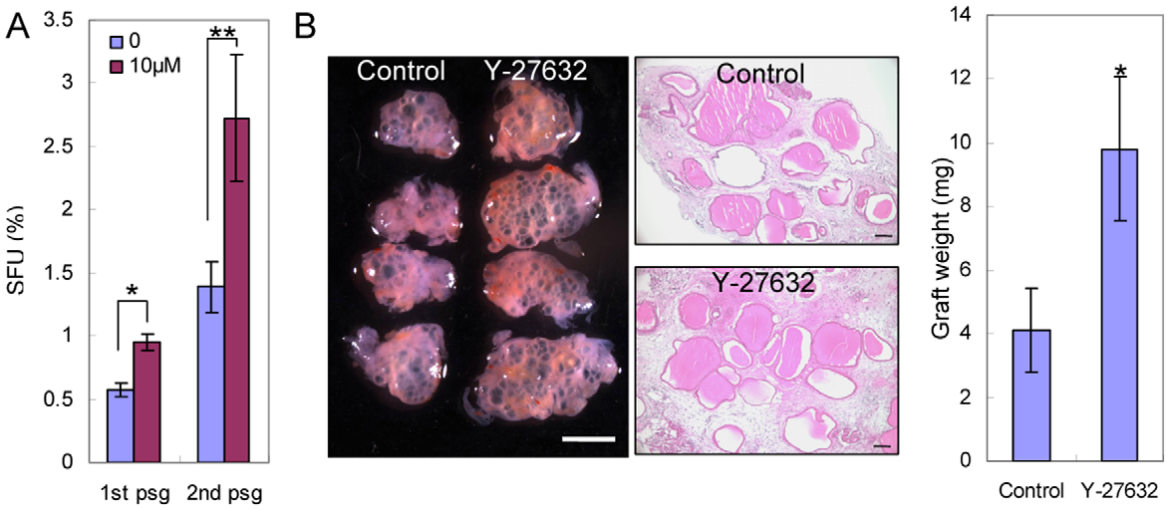
Y-27632 treatment increases cloning efficiency of prostate stem cells in the prostate sphere assay and prostate regeneration assay. (A) Bar graph shows the sphere-forming units of primary and secondary prostate sphere cultures with and without Y-27632. Error bars represent means and STD from 3 wells. * *P*<0.005, ** *P*<0.05. (B) Images show the prostate tissues regenerated from dissociated prostate cells treated with and without Y-27632 prior to prostate regeneration. White bar  = 2 mm, black bars  = 100 µm. Bar graph shows the weight of regenerated tissues. Error bars represent means and STD from 4 grafts. * *P*<0.005.

We sought to investigate whether Y-27632 treatment increases stem cell activity in those two assays. Dissociated prostate cells from 2-month-old C57BL/6 mice were seeded in 12-well plates in triplicates with or without Y-27632. Fig. 4A shows that the sphere-forming units (SFU) in the Y-27632 group is approximately 1.7 fold of that in the control group (mean SFU is 0.57±0.05% for the control group and 0.95±0.07% for the Y-27632 group, n = 3, *P* = 0.0014). Primary spheres were dissociated and passaged in the secondary culture and similar results were obtained (mean SFU in secondary culture is 1.39±0.20% for the control group and 2.72±0.50% for the Y-27632 group, n = 3, *P* = 0.012). These results demonstrated that Y-27632 treatment increases sphere-forming activity. Y-27632 is suspected to prevent apoptosis by inducing cell aggregation [26]. In the prostate sphere assay, dissociated single cells are cultured inside semi-solid matrigel, hence are immobilized. Therefore, our data also imply that the Y-27632 induced increase in stem cell activity in this assay is not due to cell aggregation.

We then investigated whether Y-27632 treatment enhances prostate-regenerating activity *in vivo* in the prostate regeneration assay. Briefly, 100,000 dissociated prostate cells from 2-month-old C57BL/6 mice were combined with 100,000 embryonic urogenital sinus mesenchymal cells and engrafted under the kidney capsules of immunodeficient CB17*^Scid/Scid^* male hosts. 10 µM Y-27632 was supplemented in all the culture media used during these procedures before the cells were grafted *in vivo*, while no Y-27632 was added in a parallel control experiment. Four grafts were set up in each group and were collected after a 6-week incubation. As shown in Fig. 4B, the regenerated tissues in the Y-27632 group weigh 2.4 fold of those in the control group (mean mass is 4.1±1.3 mg for the control group and 9.8±2.3 mg for the Y-27632 group, n = 4, *P* = 0.0049). We previously demonstrated that individual globular structures are derived from single prostate-regenerating cells [16]. There is no obvious difference in the average sizes of globular structures between the control and the Y-27632 groups (Fig. 4B). Instead, there are more globular structures in the Y-27632 group than in the control groups, suggesting that Y-27632 treatment increases the number of cells that can regenerate prostate tissues *in vivo*.

### Y-27632 mitigates dissociation-induced apoptosis of prostate cells

**Figure 5:**
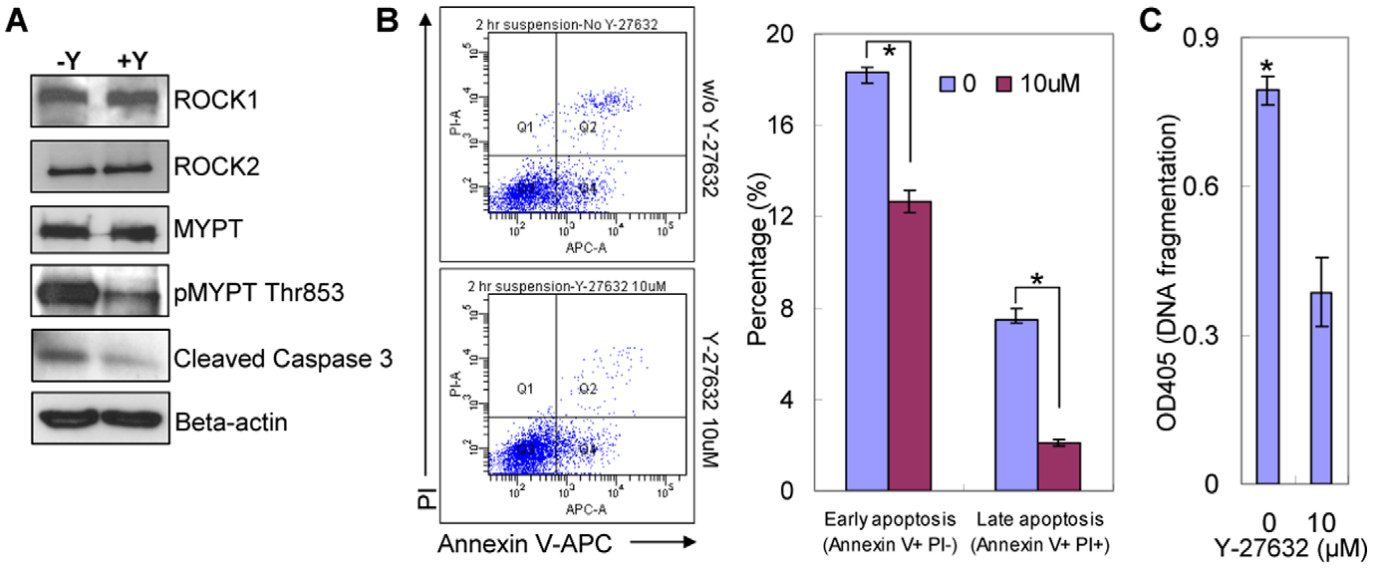
Y-27632 treatment suppresses dissociation induced apoptosis of prostate epithelial cells. (A) Western blot analyses comparing the expression of ROCK1, ROCK2, MYPT, pMYPT and cleaved caspase 3 in dissociated prostate epithelial cells treated with and without Y-27632 treatment. (B) FACS analyses with propidium iodide (PI) and Annexin V measure the percentage of apoptotic cells in dissociated prostate epithelial cells treated with and without Y-27632. Bar graphs quantify the percentage of early and late apoptotic cells. Error bars represent means and STD from 2 independent experiments. * *P*<0.005. (C) Bar graph compares the extent of DNA fragmentation in dissociated prostate epithelial cells treated with and without 10 µM Y-27632. Error bars represent means and STD from triplicates of one of the two independent experiments. * *P*<0.001.

We investigated whether Y-27632 treatment increases the cloning efficiency of prostate stem cells by suppressing dissociation-induced apoptosis. Primary prostate colony cells cultured in collagen-coated plates with or without Y-27632 were trypsinized and dissociated into single cells. Dissociated cells were cultured in suspension in ultra-low attachment plates for 2 to 4 hours with or without Y-27632 prior to subsequent analyses. Western blot analyses showed that both ROCK1 and ROCK2 are expressed in prostate colony cells and that Y-27632 treatment does not affect their expression level (Fig. 5A). One mechanism through which ROCK enhances myosin light chain (MLC) activity is to inactivate myosin light chain phosphatase (MLCP) by phosphorylating its non-catalytic subunit MYPT [3]. We showed that MYPT phosphorylation decreased significantly upon Y-27632 treatment, demonstrating that ROCK activity was efficiently inhibited.

The amount of cleaved caspase 3 is decreased in the Y-27632 treated group, indicating that Y-27632 suppresses apoptosis (Fig. 5A). Annexin V staining analysis corroborated that a higher percentage of cells underwent apoptosis in the control group than in the Y-27632 group. As shown in Fig. 5B, the percentage of the Annexin V^+^PI^−^ early apoptotic cells in the control and Y-27632 treated group are 18.4% and 12.7%, respectively; there were also more Annexin V^+^PI^+^ late apoptotic cells in the control group than in the Y-27632 treated group (7.5% versus 2.1%). Finally, an ELISA based cell death assay showed the amount of histone-complexed DNA fragments in Y-27632 treated cells was decreased by 50–60% as compared with untreated cells (Fig. 5C), further confirming that Y-27632 treatment significantly suppresses or delays dissociation-induced apoptosis of prostate epithelial cells.

### Y-27632 reduces PTEN activity and enhances AKT activity in dissociated prostate epithelial cells

**Figure 6:**
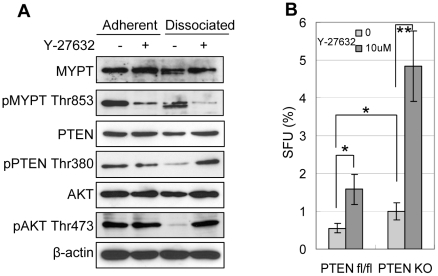
Though dissociation-induced PTEN activation is suppressed by Y-27632, it does not contribute to Y-27632 induced increase of the cloning efficiency of prostate stem/progenitor cells. (A) Western blot analyses of protein expression in prostate colony cells grown with and without 10 µM Y-27632 and in dissociated primary prostate colony cells cultured in suspension for 4 hours with and without 10 µM Y-27632. (B) Bar graph shows the sphere-forming units of WT and PTEN KO prostate cells in the presence and absence of Y-27632. Error bars represent means and STD from 3 wells. * *P*<0.05, ** *P*<0.005.

The PI3K-AKT signaling pathway plays an important role in prostate epithelial cell survival, proliferation and transformation [27], [28]. Reciprocal crosstalk between the PI3K-AKT and the RhoA-ROCK signaling pathways has been documented [29], [30], [31]. We sought to investigate whether the activities of PTEN and AKT were affected upon cell dissociation and/or inhibition of ROCK activation. Primary prostate colony cells were dissociated and cultured in ultra-low attachment plates for 4 hours with or without Y-27632. Western blot analyses were performed using lysates from prostate colony cells that were freshly dissociated and those that were cultured in suspension for 4 hours after dissociation. Western Blot analysis confirms that ROCK activity was efficiently inhibited by Y-27632 in both adherent and suspended cells since MYPT phosphorylation was decreased significantly (Fig. 6A). Previously, Vazquez *et al* demonstrated that PTEN phosphorylation at the C-terminus including Thr380 suppresses its phosphatase activity [32], [33], [34]. Fig. 6A shows that the expression levels of PTEN, Thr380 pPTEN, AKT and Thr472 pAKT were comparable in adherent prostate epithelial cells cultured with and without Y-27632. After dissociated cells were cultured in suspension for 4 hours, Thr380 phosphorylated PTEN and Thr472 phosphorylated AKT were significantly decreased, while the expression levels of total PTEN and AKT were not dramatically altered. In contrast, the activities of PTEN and AKT in the dissociated cells cultured in suspension in the presence of Y-27632 were comparable to the cells prior to dissociation. Overall, these data demonstrated that inhibiting ROCK activity suppresses dissociation-induced PTEN activation.

### Y-27632 increased cloning efficiency of prostate stem cells is not through modulation of PTEN activity

To determine whether Y-27632 suppresses dissociation induced apoptosis partly through inhibition of PTEN activity, we investigated whether Y-27632 treatment would alter the cloning efficiency of PTEN null prostate stem/progenitor cells using the prostate sphere assay. We previously demonstrated that there is an expansion of prostate basal epithelial cells in a PB-Cre;Pten^fl/fl^ mouse model, in which PTEN is specifically deleted in both the prostate basal and luminal epithelial cells [35], [36]. Dissociated prostate cells were prepared from 8-week-old PB-Cre;Pten^fl/fl^ mice and control Pten^fl/fl^ mice. Cells from each group were seeded at a density of 5000 cells per well in 12-well plates in triplicates with or without Y-27632. Fig. 6B shows that the SFU of PTEN KO cells is 1.8-fold of that of the control WT cells, corroborating our previous observation that basal cells expanded in PTEN KO mouse prostates (mean SFU is 0.55±0.13% for the WT group and 0.99±0.22% for PTEN KO group, n = 3, *P* = 0.042). However, Y-27632 treatment increased the SFU of both the wild type cells and the PTEN KO cells by 2.86 and 4.88 fold, respectively (mean SFU is 1.58±0.40% for the WT group and 4.85±0.93% for PTEN KO group in the presence of Y-27632). These data demonstrate that modulation of PTEN activity does not play a major role in the Y-27632 induced increase of cloning efficiency of prostate stem/progenitor cells.

### Simultaneous down-regulation of ROCK1 and ROCK2 expression slightly increases the cloning efficiency of prostate colony cells

**Figure 7:**
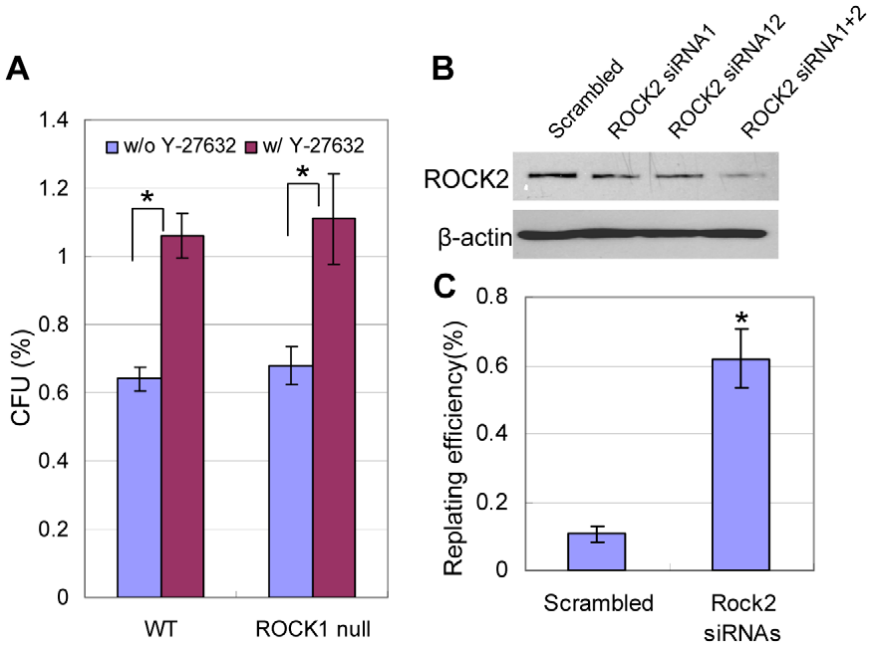
Simultaneous down-regulation of ROCK1 and ROCK2 expression increases the cloning efficiency of prostate colony cells. (A) Bar graph compares the colony-forming activity of WT and ROCK1 KO prostate colonies grown in 12-well collagen-coated plates with or without Y-27632. *P<0.005, **P<0.05. (B) Western Blot analysis of ROCK2 expression in ROCK1 null prostate colony cells treated with siRNAs targeting ROCK2 or the scrambled siRNA. (C) Bar graph compares the replating efficiency of the scambled siRNA- and ROCK2 siRNAs-treated ROCK1 null prostate colony cells. *P<0.001.

Y-27632 has been shown to inhibit activities of kinases other than ROCKs [37]. We sought to investigate whether suppressing ROCK kinase activities contributes substantially to the increased cloning efficiency of prostate stem cells by Y-27632. Prostate epithelial cells express both ROCK1 and ROCK2 (Fig. 5A). ROCK1 knockout mice are viable [19] and their prostates develop normally (data not shown). Y-27632 treatment increases the CFUs of prostate epithelial cells from both the wild type and ROCK1 knockout mice to the same extent (Fig. 7A), suggesting that ROCK2 plays a redundant function. To eliminate both ROCK kinases in prostate epithelial cells, ROCK1 null primarily prostate cells were transfected with two different siRNAs that target ROCK2. These siRNAs are capable of suppressing ROCK2 partially when used in combination (Fig. 7B). Figure 7C shows that when both ROCK kinases were knocked down, 0.6% prostate colony cells can be replated while less than 0.1% of the ROCK1 null mouse cells can. This result demonstrates that suppressing expression/activity of both ROCK kinases is sufficient to increases the cloning efficiency of prostate colony cells. However, the replating efficiency is still much lower as compared to 7% when Y-27632 is present. Additionally, the secondary colonies are tiny and usually contain less than 20 cells. This is probably due to the incomplete knockdown of ROCK2 by siRNAs. Or alternatively, other targets of Y-27632 may play a role.

## Discussion

Our study demonstrates a role of the RhoA/ROCK signaling pathway in the apoptosis of murine prostate stem/progenitor cells upon dissociation from their environmental cues. A recent study reported that Y-27632 treatment also increases the yield of *in vitro* culture of prostate epithelial cells from human prostate specimens [38]. Altogether, our study and previous studies on embryonic stem cells and small intestinal stem cells suggest that dissociation-induced RhoA/ROCK-mediated apoptosis is a common feature of embryonic and somatic stem cells with an epithelial phenotype.

### Distinctive roles of PTEN and RhoA/ROCK in regulating stem cell activity

PTEN activity has been shown previously to modulate stem cell self-renewal and proliferation. For example, PTEN confers a negative effect on neural stem cell self-renewal and proliferation [39], [40]. Another study showed that knocking down PTEN increases the cloning efficiency of normal mammary gland epithelial stem cells using a mammosphere assay [41]. We previously demonstrated that PTEN null LSC basal/stem cells form prostate spheres at a higher efficiency than the control wild type LSC cells (approximately 1.5 fold) [35], suggesting that loss of PTEN activity increase *in vitro* proliferation of prostate stem/progenitor cells. We showed in this study that Y-27632 suppresses cell dissociation induced apoptosis and is capable of increasing cloning efficiency of both wild type and Pten null prostate stem/progenitor cells. These data suggests that the RhoA-ROCK and PTEN-AKT signaling pathways mediate distinctive signaling regulating stem cell survival and proliferation although there is a crosstalk between them.

### Regulation of PTEN activity by the RhoA/ROCK signaling pathway in prostate cancer

Several studies have demonstrated that RhoA activation enhances PTEN activity and suppresses AKT activation in fibroblast cells [42], neutrophils [31] and endothelial cells [43]. Li Z *et al* showed that ROCK can directly phosphorylate PTEN at Ser229-Thr223 and Thr319-Thr321 [31]. It remains unresolved how PTEN Thr380 phosphorylation is regulated by the RhoA/ROCK signaling pathway. Casein Kinase 2 and GSK-3β are two candidate kinases that phosphorylate PTEN at Thr380 [44]. It is possible that ROCK negatively regulates the activities of these two kinases. Alternatively, phosphorylation of PTEN at Ser229-Thr223 and Thr319-Thr321by ROCK facilitates removal of Thr380 phosphorylation or prevents Thr380 phosphorylation. Interestingly, Y-27632 does not affect PTEN activity in adherent cells (Fig.6A), suggesting that ROCK only or mainly modulates PTEN activity when cells are dissociated from their environmental cues.

Though regulation of PTEN activity by the RhoA/ROCK signaling pathway does not seem to contribute to the *in vitro* survival of normal prostate stem cells, it has been shown to play an essential role in Ras-induced tumorigenesis of lung cancer cells [45]. Ras activation causes upregulation of an oncoprotein Gankyrin. Gankyrin suppresses RhoA activation and inhibits ROCK activation, which in turn suppresses PTEN activity and leads to prolonged AKT activation. In prostate cancer, though activating mutations in Ras are not frequently reported, loss of DAB2IP, a Ras GTPase-activating protein (RasGAP), has been closely associated with aggressive metastatic prostate cancer [46], [47]. Decreased RhoA activity has been shown to induce breakdown of basement membrane and facilitate epithelial-mesenchymal transition during gastrulation [48]. Therefore, it is natural to postulate that attenuation of ROCK activity and PTEN activity may play a role in Ras activation-induced prostate cancer progression and especially metastasis.

### The lineage status of prostate colony cells and prostate sphere cells

Previously it has been shown that freshly FACS sorted LSC basal cells are capable of forming glandular structures that consist of all three types of prostate epithelial cells [14], [15]. This demonstrates that those cells possess the capacity of stem cells for multi-lineage differentiation. In contrast, after LSC cells were cultured in the prostate sphere assay, the capability of the resulting prostate sphere cells to differentiate into multiple lineages *in vivo* dropped dramatically [17]. We also tested whether the prostate colony cells are capable of differentiating into all three prostate epithelial cell lineages and can form glandular structures *in vivo*. Our results showed that even when 2×10^5^ prostate colony cells were used for *in vivo* regeneration, we can only occasionally detect 1 or 2 tubular structures containing basal and luminal epithelial cells (data not shown). These observations suggest that upon short-term culture LSC cells lose their multipotent stem cell activity. Since only a small fraction of prostate sphere cells and prostate colony cells are capable of replating to respectively form secondary spheres and colonies, they may represent a committed basal progenitor population, or transit-amplifying cells [49]. Understanding the signaling that mediates the loss of multipotency will provide insights into how prostate stem cells are maintained *in vivo*.
